# A revision of the genus *Psammogorgia* Verrill, 1868 (Cnidaria, Anthozoa, Octocorallia) in the tropical eastern Pacific Ocean

**DOI:** 10.3897/zookeys.961.54846

**Published:** 2020-08-19

**Authors:** Odalisca Breedy, Hector M. Guzman

**Affiliations:** 1 Centro de Investigación en Estructuras Microscópicas, Centro de Investigación en Ciencias del Mar y Limnología, Escuela de Biología, Universidad de Costa Rica. P.O. Box 11501-2060, Universidad de Costa Rica, San José, Costa Rica Smithsonian Tropical Research Institute Panama Panama; 2 Smithsonian Tropical Research Institute, P.O. Box 0843-03092, Panama, Republic of Panama Universidad de Costa Rica San José Costa Rica

**Keywords:** Biodiversity, gorgonians, key to plexaurid genera, octocoral, Plexauridae, taxonomic review, taxonomy

## Abstract

The species of the genus *Psammogorgia* Verrill, 1868 from the shallow waters of the tropical eastern Pacific were mainly described from 1846 to 1870. Very few contributions were published subsequently. Recently, the genus was revisited with the addition of two new species. However, a comprehensive generic study is still missing for the eastern Pacific. *Psammogorgia* is characterised by having axes cores without mineralisation, mainly coarse irregular spindles and thorny, leafy or tuberculate clubs coenenchymal sclerites and the anthocodial armature with distinct collaret and points arrangements. Herein a taxonomic revision of the genus is presented based on type material which was morphologically analysed and illustrated using optical and scanning electron microscopy. Comparative character tables are provided for comparison among species in the genus, along with a taxonomic key. Moreover, the taxonomic status of each species was analysed. The genus *Psammogorgia* comprises six valid species and two varieties, and three lectotypes and a new combination are proposed to establish the taxonomic status of these species.

## Introduction

Seven genera in the family Plexauridae have been reported for the tropical eastern Pacific: *Muricea*, Lamouroux, 1821; *Thesea* Duchassaing & Michelotti, 1860; *Swiftia* Duchassaing & Michelotti, 1864; *Heterogorgia* Verrill, 1868a; *Psammogorgia* Verrill, 1868a; *Adelogorgia* Bayer, 1958 and *Chromoplexaura* Williams, 2013. Previous taxonomic reviews of Plexauridae for the region dealt with *Heterogorgia* Verrill, 1868a and *Muricea* Lamouroux, 1821 (Breedy and Guzman 2011, 2015, 2016).

The genus *Psammogorgia* Verrill, 1868a was established by Verrill to place a species previously assigned to the genus *Echinogorgia* Kölliker, 1865 (*E.arbuscula* Verrill, 1866), which was subsequently named *Psammogorgiaarbuscula* (Verrill, 1868a). Later, Verrill (1868b) properly described *P.arbuscula* and two of its varieties: (Psammogorgiaarbusculavar.pallida Verrill, 1868, and P.arbusculavar.dowii Verrill, 1868), and two other species (*Psammogorgiagracilis* Verrill, 1868 and *Psammogorgiateres* Verrill, 1868). In his review, Verrill (1868b) also included material of *Gorgoniafucosa* Valenciennes, 1846, which was collected during the French expedition ‘Voyage autour du monde sur la frégate la Vénus’.

From 1868 to 1951 more species were described within the genus *Psammogorgia* from different regions and bathymetric ranges (Studer 1878; Ridley 1888; Studer 1894; Nutting 1909; Thomson and Simpson 1909; Thomson 1911; Kükenthal 1919; Stiasny 1935, 1951). Some of these species have been studied and placed in different genera, while the taxonomic status of others remains uncertain and in need of revision. Nutting (1909) proposed three species, *Psammogorgiasimplex* Nutting, 1909, *Psammogorgiaspauldingi* Nutting, 1909, and *Psammogorgiatorreyi* Nutting, 1909 from California, which are presently placed in the genus *Swiftia* Duchassaing & Michelotti, 1864. Stiasny (1951) described *Psammogorgiadigueti* Stiasny, 1951 from Canal San Lorenzo, Gulf of California, which according to Bayer (1958) is a species of the genus with “ the size of sclerites given by Stiasny being exceptionally small.”

The status of most species of the genus *Psammogorgia* is uncertain because the previous authors did not designate holotypes and the illustrations of specimens and sclerites in old publications are mostly insufficient for proper species identification. Additionally, some species have been described from one to few specimens or fragments, while their type material is lost to science or their location unknown. According to Bayer (1961) without an accurate knowledge of the type material, no clear concept of genera or species can exist.

Breedy and Guzman (2014, 2020) revisited the genus *Psammogorgia* and described two species: *Psammogorgiahookeri* Breedy & Guzman, 2014 from Perú, and *Psammogorgiapax* Breedy et al., 2020 from Panamá. However, a comprehensive review with the original type material of this genus is necessary to establish the status of the species. Herein, we present a taxonomic revision of the genus *Psammogorgia* in the tropical eastern Pacific based on type material. This research represents the seventh and last review in a series proposed to evaluate the genera of gorgonians historically reported from the shallow eastern Pacific waters.

**Acronyms**

**MCZ**Museum of Comparative Zoology, Harvard University, Boston, USA.

**MNHN** Muséum national d’Histoire naturelle, Paris, France.

**NMNM / USNM** National Museum of Natural History, Smithsonian Institution, Washington, USA.

**YPM** Yale Peabody Museum of Natural History, New Haven, USA.

## Material and methods

The type specimens used in this study were analysed during visits to museums or acquired on loan from the **MCZ, MNHN, NMNM**, and **YPM**. For the species *Psammogorgiafucosa* (Valenciennes, 1846), the only type material available is a sclerite slide found in the MCZ. Depth of collection of the type specimens was not recorded; however, most of the types collected by F.M. Bradley were obtained by pearl divers between 8 and 12 m in depth (Verrill 1868b).

The taxonomic identification and description of the octocorals was based on external morphology: shape, size and colour of the colonies, and calyx structures, as well as on internal morphology: sclerites content, dominance, shape, size and arrangement. Terminology used in this study mostly follows Bayer et al. (1983). For microscopic study, fragments of the tips of the colonies were treated with 5% sodium hypochlorite to dissociate sclerites from the tissues. The structures were washed several times in distilled water and dehydrated with 100% ethanol and posteriorly dried in the oven (Breedy and Guzman 2002). For old specimens in bad conditions it was difficult to clean the sclerites. These samples were treated with hydrogen peroxide to remove remains of organic matter, but most sclerites from these samples were still dirty as shown by the Scanning Electron Microscope (**SEM**) micrographs. Notes on the colours of the colonies and sclerites based on dry type material and literature reports were taken, considering that colours are stable and persist after fixation of the *Psammogorgia* specimens.

In order to prepare the sclerome for imaging and measurements, different microscope preparations were made. For optic microscopy, sclerites were mounted in water or glycerine and photographed with an Olympus LX 51 inverted microscope. For SEM, sclerites were mounted on SEM stubs by double stick carbon tape and silver paint bridges between the tape and the stubs were made to increase the electronic conduction. The samples were then sputter-coated with gold, 30–60 nm layer, in an Eiko IB-5 Ion Coater and the pictures were obtained using a Hitachi SEM S-3700N. Unsorted optic microscope micrographs reveal colour details and sclerites composition while the SEM illustrations show details and sculpture of the sclerites. Not all sclerite types of a species are presented in the SEM figures. Measurements of the sclerites were obtained from the SEM images, and for *P.fucosa* from the optical micrographs the length of the sclerites was measured from one tip to the other and the width was taken from the most distant points across the sclerites, reporting the largest sizes found in the samples. Because type material was generally in bad condition, the anthocodial sclerite arrangement at the base of the polyps was not described in some cases. The diameter of the branches, branchlets, and stems was noted, taking the length of the calyces into account.

Designation of lectotypes was done for three species with unclear identity described by either Verrill or Valenciennes without type designation. Lastly, data on geographical distributions are based on our personal collections (Museo de Zoología, Universidad de Costa Rica, Naos Laboratory, Smithsonian Tropical Research Institut, Panamá, **STRI**), museum catalogues and published monographs.

## Taxonomy

### Key to plexaurid genera presently reported from the tropical eastern Pacific

**Table:** 

1	Coenenchyme contains massive unilateral spinous sclerites. Polyps retract into shelf-like or tubular calyces	***Muricea***
–	Coenenchyme does not contain unilateral spinous sclerites. Polyps do not retract into shelf-like or tubular calyces	**2**
2	Calyces with lobed rims armed with strongly projecting thorns forming a bristling barricade around calycular apertures. Axis’s cores with organic fibres mineralised with carbonate hydroxylapatite	***Heterogorgia***
–	Calyces without lobed rims armed with strongly projecting thorns forming a bristling barricade around calycular apertures. Axis’s cores with organic fibres non-mineralised with carbonate hydroxylapatite	**3**
3	External coenenchyme with characteristic large rugose plates having the inner side with low composite warts, and the outer side with wide lobes	***Thesea***
–	External coenenchyme without characteristic large rugose plates having the inner side with low composite warts, and the outer side with wide lobes	**4**
4	External coenenchyme with conspicuous double disk sclerites with one side expanded in longitudinal crests with various degrees of ornamentation	***Adelogorgia***
–	External coenenchyme without conspicuous double disk sclerites, with one side expanded in longitudinal crests with various degrees of ornamentation	**5**
5	Coenenchymal sclerites mainly thin, sharp spindles with or without fused tubercles in incomplete disks. Anthocodial armature with a few bar-like rods transversely arranged not forming distinct collaret and points	***Swiftia***
–	Coenenchymal sclerites without thin, sharp spindles fused in incomplete disks. Anthocodial armature forming distinct collaret and points	**6**
6	Coenenchymal sclerites mainly coarse, irregular spindles and thorny, foliate or tuberculate clubs	***Psammogorgia***
–	Coenenchymal sclerites mainly radiates and spindles, without foliate or tuberculate clubs	***Chromoplexaura***

### Systematics

#### Class Anthozoa Ehrenberg, 1834

**Subclass Octocorallia Haeckel, 1866**

**Order Alcyonacea Lamouroux, 1816**

**Family Plexauridae Gray, 1859**

##### Genus Psammogorgia

Taxon classificationAnimaliaAlcyonaceaPlexauridae

Verrill, 1868

ACCA435D-0E3E-5971-AC95-1C697C739469

Psammogorgia Verrill, 1868a: 414; Verrill 1868b: 414; Studer 1887: 60; Wright 1889: lix; Nutting 1909: 719; Nutting 1910: 16; Kükenthal 1919: 234–236, 905; Kükenthal 1924: 106; Bayer 1956: F212; Bayer 1958: 43; Harden 1979: 114; Bayer 1981: 925; Breedy and Guzman 2014: 494; Breedy et al. 2020: 171–172.

###### Type species.

*Echinogorgiaarbuscula* Verrill, 1866 by subsequent designation (Verrill 1868b).

###### Diagnosis.

Colonies bushy to flabellate. Branching lateral, dichotomous, irregularly dichotomous, or subpinnate. Branches round or slightly flattened. Axis horny, chambered central core filled with organic non-mineralised fibres. Calyces on all sides of branches, flat, slightly raised or prominent. Polyp apertures slit-like or swollen. Anthocodial sclerites mostly large, elongated, warty, spinose or slender spindles, with or without median waist, in collaret and points arrangements at base of tentacles. Sclerites of coenenchyme thick, warty spindles; radiates, and crosses. Clubs warty or foliate-like with variation of those types mostly present at calyx rims and external coenenchyme. Colony colours dark red, red, orange, pink and white. Sclerites colours red, pink, orange, yellow, various hues of these, and/or colourless.

###### Distribution.

The genus has been reported from the eastern Pacific, Californian province, the Indian Ocean and the north Atlantic.

###### Remarks.

**Figure 1.**
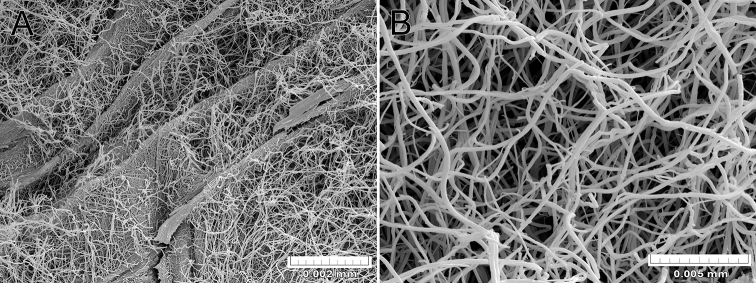
*Psammogorgiaarbuscula* (Verrill, 1866) STRI 269 **A** central core chambers filled with organic non-mineralised fibres **B** detail of organic non-mineralised fibres.

Axes analysis of the species of *Psammogorgia* show chambered central cores filled with organic non-mineralised fibres (e.g., Fig. 1).

##### Psammogorgia arbuscula

Taxon classificationAnimaliaAlcyonaceaPlexauridae

(Verrill, 1866)

2AD15E96-D0BA-5222-A839-AB091F10895B

Figures 2 , 3 , 4

Echinogorgia arbuscula Verrill, 1866: 329.
Psammogorgia arbuscula Verrill, 1868a: 414; 1868b: 414–415; not Nutting 1909: 719–720; Nutting 1910: 16; Kükenthal 1919: 236–237; Kükenthal 1924: 107; Bayer 1958: 44; Harden 1979: 114–116.
Psammogorgia arbuscula typica Kükenthal, 1924: 107.

###### Type material.

***Lectotype*** (designated herein). YPM 573, dry, Pearl Islands, Gulf of Panamá, Panamá, F.H. Bradley, 1866–1867, no additional data.

***Paralectotypes*.**YPM 573 a-h; MCZ 425B, MCZ 573 (part of YPM 573), MCZ 727, 728A-B (4916=YPM 1577), MCZ 4017–4019, MCZ 4021–4022, MCZ 4024, MCZ 4998 (=MCZ 728) same data as the lectotype. MCZ 7009, dry, Nicoya Gulf, Costa Rica, collected by pearl divers, J.A. Mc. Neil, 1866–1867, no additional data.

###### Type locality.

Pearl Islands, Panamá.

###### Diagnosis.

Colonies bushy, irregularly dichotomous. Stems short, slightly flattened, one to several stems emerging from a common holdfast. Branches and branchlets thin, rounded with long free ends in large colonies. Holdfasts encrusting with a thin layer of coenenchyme often with polyps. Coenenchyme of branches moderately thick and granulose. Coenenchymal sclerites: irregular spindles with acute or bifurcated ends, up to 0.30 mm long; warty and irregular radiates up to 0.13 mm long and some crosses. Calyces prominent and swollen, all around the branches, mostly closely placed in two or three longitudinal rows on each side of the branches. Calyces with thorny and irregular spindles and wart-clubs, up to 0.19 mm long, around the calyx rim. Anthocodial spindles up to 0.26 mm long, in collaret and points arrangements. Sclerites red and orange. Dry colonies red to red-orange, dark red when alive. Polyps bright yellow when alive.

###### Description.

**Figure 2.**
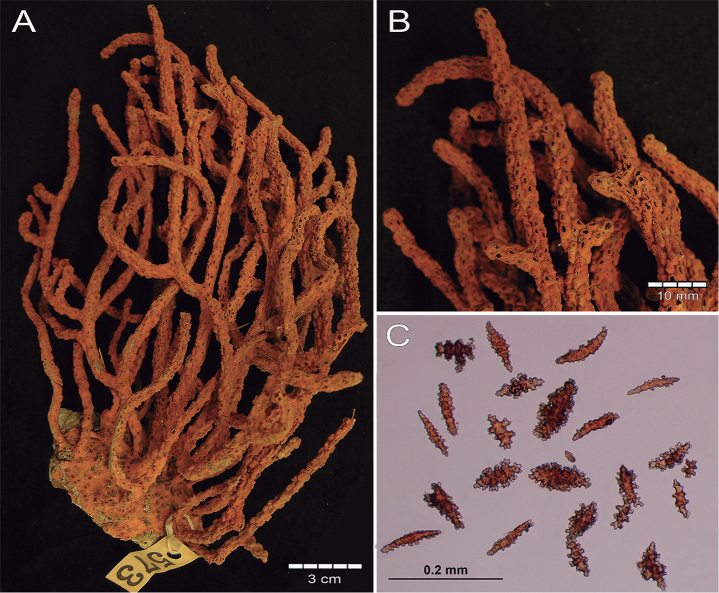
*Psammogorgiaarbuscula* (Verrill, 1866) YPM 573 **A** colony **B** detail of branches **C** coenenchymal and anthocodial sclerites.

**Figure 3.**
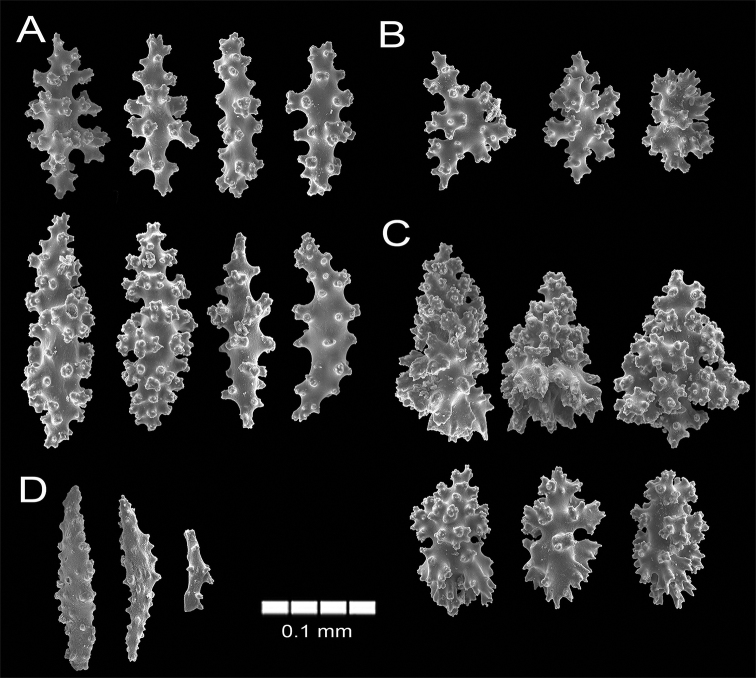
*Psammogorgiaarbuscula* (Verrill, 1866) YPM 573 **A** spindles **B** radiates **C** wart clubs **D** anthocodial spindles.

(see also Verrill 1866, 1868b; Bayer 1956). The lectotype is a bushy, irregularly dichotomous dry colony, 12 cm long and 9.5 cm wide. The colony is of a red orange colour (Fig. 2A, B). Nine stems arise from an oval encrusting holdfast which is ~ 4.5 cm in diameter (Fig. 2A). The holdfast is covered by a thin layer of coenenchyme with polyps. Most of the stems are 3.0–3.2 mm in diameter bifurcating a few mm above the base, two of them raising up to 2 cm before subdividing in several branches 2–4 mm in diameter including calyces. The colony branches up to eight times. The branches emerge at angles of 45–90°, ascending parallel and slightly curved at the end. Terminal branchlets are 2.5–10 mm long (Fig. 2A). The calyces are with rounded somewhat tapered tips. Calyces are closely arranged around the branches, mostly in 2–3 longitudinal rows on each side of the branches; somehow in quincunx (arrangements of five) as Verrill (1868b) mentioned (Fig. 2B). The calyces are prominent, up to 1 mm tall and around 2 mm diameter, composed of eight marginal swollen lobes around the polyp apertures, which is evident when polyps are withdraw or in dry condition (Fig. 2B). Calyces present a concentration of thorny, irregular spindles and wart-clubs around the borders. The coenenchyme is moderately thick, granulose and brittle in the dry lectotype. Coenenchymal sclerites are dark red, red, orange and of lighter hues (Fig. 2C), and of different forms: irregular spindles with acute or bifurcated ends, some being slightly curved (Figs 2C, 3A), 0.14–0.20 mm long and 0.06–0.07 mm wide. Warty radiates are 0.07–0.13 mm long and 0.065–0.085 mm wide (Fig. 3B); and some warty crosses up to 0.11 mm by 0.1 mm. Wart-clubs are 0.09–0.18 mm long and 0.049–0.12 mm wide, variable in form and with a larger end expanded and covered with thorny warts (Fig. 3C). They are concentrated at the calyx rims and the base of the anthocodia. The anthocodial armature is well developed. It is composed of spiny spindles arranged in a collaret and points, 0.13–0.17 mm long (0.20–0.26 mm long according to Verrill (1868b)) and 0.02–0.045 mm wide (Fig. 3D); its flat spindles are with small tubercles and scattered warts.

###### Variability.

**Figure 4.**
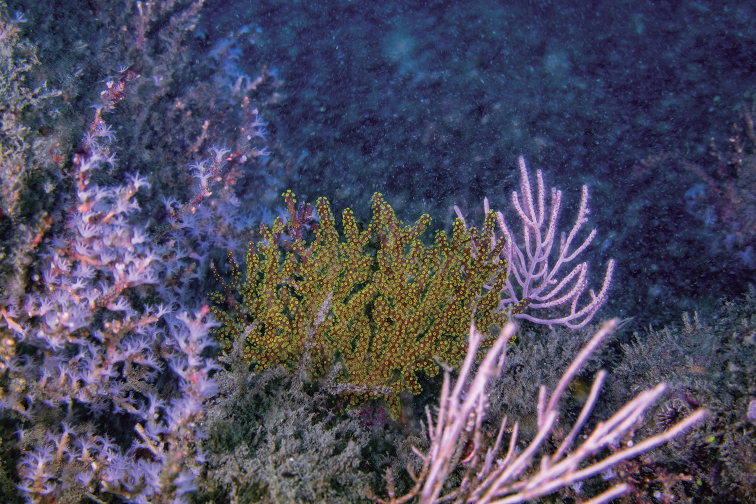
*Psammogorgiaarbuscula* (Verrill, 1866). A *In situ* colony, 13 m deep, Rocas Corcovado, Osa Peninsula, Costa Rica. Photograph: Manu San Felix, National Geographic Pristine Seas.

Most of the type material of the form typica of *P.arbuscula* is constituted of small colonies 5–15 cm long and 3–7 cm wide or fragments of colonies, the largest specimen being MCZ 7009 (28 cm long and 20 cm wide), with unbranched ends up to 15 cm long. Stems can reach up to 4 mm diameter, branches up to 3.5–3.8 mm in diameter and branchlets up to 2.0–2.6 mm in diameter. The sclerites content is consistent among the types. When alive, the colonies are dark red and the polyps are bright yellow (Verrill 1868b) (Fig. 4).

###### Distribution.

Tropical eastern Pacific: Panamá, Costa Rica, Ecuador, México and El Salvador.

###### Remarks and comparison.

The *Psammogorgiaarbusculatypica* is different in calyx structure, size of sclerites and colour from the varieties P.arbusculavar.dowii and P.arbusculavar.pallida (see Tables 1, 2). These other varieties lack the prominent and swollen calyces present in *P.arbusculatypica*. *Psammogorgiagracilis* and *Psammogorgiahookeri* have prominent calyces however, there are many other differences that separate them from *P.arbuscula* (Tables 1, 2). *Psammogorgiagracilis* has thinner and longer branchlets as well as shorter spindles, longer wart clubs, and shorter anthocodial sclerites (Tables 1, 2). *Psammogorgiahookeri* has smaller bushy colonies with a typical coral red colour, different from the larger colonies of *P.arbuscula*. In general, *P.hookeri* has star-like sclerites absent in the later, and smaller sclerites than in *P.arbuscula*.

**Table 1.** Comparative features of *Psammogorgia* colonies from the tropical eastern Pacific, according to analyses of type material from museums (YPM, MCZ, MNHN), and taxonomic descriptions by Verrill (1868b, 1870) and Bayer (1958). Diameter of the branches includes calyces. Measurements in millimetres.

Species	Colony colour	Colony shape and branching pattern	Maximum # branching	Length of terminal branchlets	Diameter of branchlets	Branch anastomosis	Calyx of branchlets	Presence of swollen calyx rims	Calyx arrangement at branchlets
*P.arbuscula* (Verrill, 1866)	dark red	bushy, irregularly dichotomous	8	2.5–15	2–4	absent	prominent	yes	close
P.arbusculavar.dowii Verrill, 1868b	dark red	*flabellate, dichotomous	2	6–35	2	absent	slightly raised	no	sparse
P.arbusculavar.pallida Verrill, 1868b	yellowish	flabellate, irregularly dichotomous	15	40	2–3	absent	flat/ slightly raised	no	sparse
*P.fucosa* Valenciennes, 1846	reddish	bushy, irregularly dichotomous	12	12.7–25.4	3–4.5	absent	flat	no	sparse
*P.gracilis* Verrill, 1868	red	*flabellate, irregular dichotomous	9	60	1.5–1.6	absent	prominent	yes	close
*P.hookeri* Breedy & Guzman, 2014	coral red	bushy, irregularly dichotomous	8	10–15	2–2.5	absent	prominent	yes	close
*P.pax* Breedy et al. 2020	white	flabellate, irregularly dichotomous	20	10–125	3–4	present	slightly raised	no	sparse
*P.teres* Verrill, 1868	red orange	bushy, irregularly dichotomous	12	5–60	3–5	absent	flat	no	sparse

*Data from Verrill (1868b): syntype colony is small or a fragment.

**Table 2.** Comparative features of sclerites of *Psammogorgia* species in the tropical eastern Pacific Ocean according to an analysis of type material from museums (YPM, MCZ, MNHN) and taxonomic descriptions by Verrill (1868b, 1870), Bayer (1958), Breedy and Guzman (2014), and Breedy et al. (2020).

Species	Spindle length (mm)	Wart club length (mm)	Radiates length (mm)	Anthocodials length (mm)	Colour of coenenchymal sclerites	Colour of anthocodial sclerites
*P.arbuscula* (Verrill, 1866)	0.14–0.30	0.09–0.18	0.07–0.13	0.12–0.26	dark red, red, orange	red, orange
P.arbusculavar.dowii Verrill, 1868	0.14–0.21	0.11–0.18	0.13–0.15	0.12–0.20	dark red, red, orange	red, orange
P.arbusculavar.pallida Verrill, 1868	0.15–0.23	0.11–0.16	0.08–0.16	0.11–0.23	pale pink, colourless	orange red
*P.fucosa* Valenciennes, 1846	0.10–0.22	0.10–0.18	0.09–0.11	0.10–0.21	red, pink, colourless	red
*P.gracilis* Verrill, 1868	0.12–0.24	0.11–0.25	0.08–0.10	0.11–0.20	red, orange, yellow	orange, pale yellow
*P.hookeri* Breedy & Guzman, 2014	0.12–0.19	0.11–0.16	0.09–0.11	0.10–0.18	coral red, reddish	yellowish, pale pink
*P.pax* Breedy et al. 2020	0.21–0.24	0.13–0.33	not found	0.21–0.26	colourless	orange
*P.teres* Verrill, 1868	0.11–0.20	0.07–0.16	0.07–0.14	0.13–0.26	red, orange, colourless	pale yellow, colourless

Bayer (1958) treated specimen MCZ 4022 as the holotype for the species however, Verrill did not designate a holotype. Verrill´s 1866 original description of *Echinogorgiaarbuscula* is general, and he did not describe specimen MCZ 4022 specifically. We consider specimen YPM 573 more representative of the species and designate this as the lectotype to clearly establish the taxonomic status of *P.arbuscula*.

##### Psammogorgia arbuscula var. dowii

Taxon classificationAnimaliaAlcyonaceaPlexauridae

Verrill, 1868

8FFB2F24-43E1-5FD8-89BC-6B1423B95F3F

Figures 5 , 6

Psammogorgia arbuscula var. dowii Verrill, 1868b: 415; Kükenthal 1919: 237; Harden 1979: 117.
Psammogorgia arbuscula dowii Kükenthal, 1924: 107.

###### Type material.

***Syntypes***: YPM 1787, dry, Pearl Islands, Panamá, F.H. Bradley, 1866–1867, no additional data. YPM 8684 (fragments, mixture of species), not P.arbusculavar.dowii, dry, Pearl Islands, Panamá, F.H. Bradley, 1866–1867, no additional data.

###### Description.

**Figure 5.**
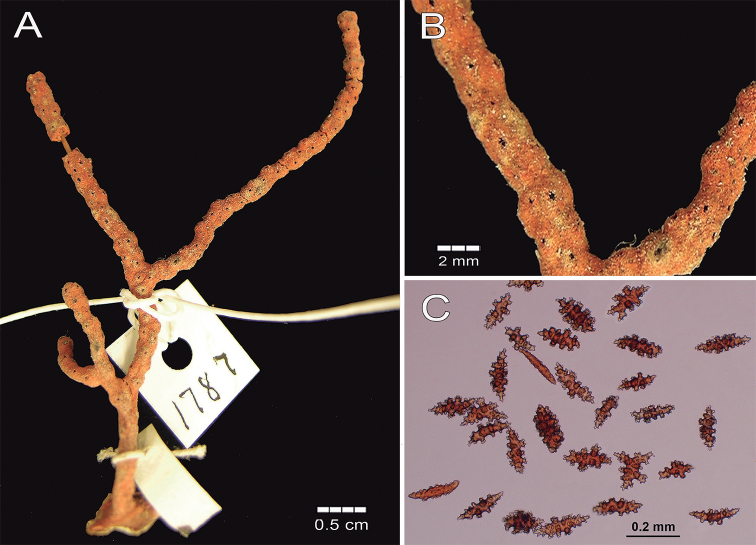
Psammogorgiaarbusculavar.dowii Verrill, 1868. YPM 1787 **A** colony **B** detail of branches **C** coenenchymal and anthocodial sclerites.

**Figure 6.**
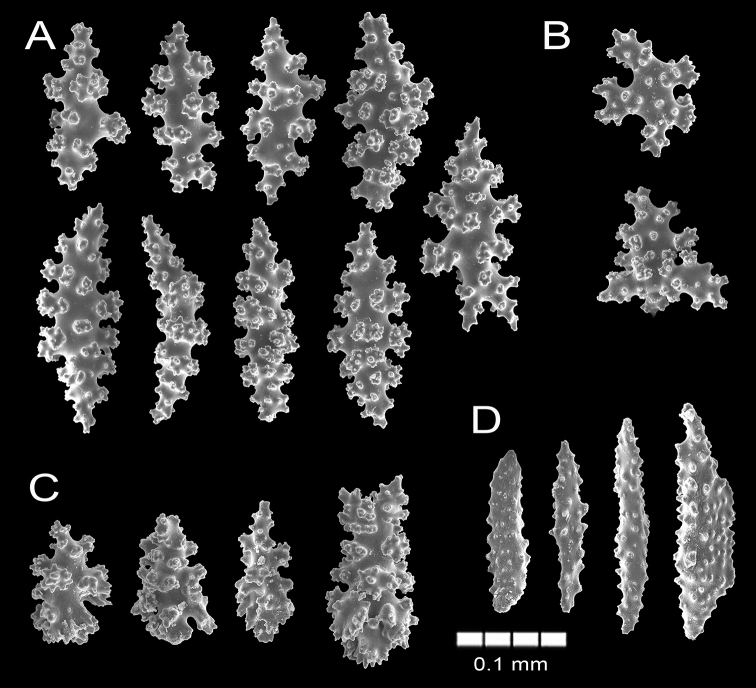
Psammogorgiaarbusculavar.dowii Verrill, 1868. YPM 1787 **A** spindles **B** radiate and cross **C** wart clubs **D** anthocodial spindles.

The syntype YPM 1787 is a small, 5.8 cm long dark red colony of two dichotomous branches. A 1.1 cm long stem arises from an oval holdfast ~ 1 cm in diameter (Fig. 5A). The holdfast is covered by a thin layer of coenenchyme without polyps. The stem is 2.0 mm in diameter and bifurcates, subdividing in two branchlets up to 3.5 cm long. The branchlets are of the same diameter as the stem, with rounded tips. The branchlets bifurcate at angles of 45°, ascending parallel and are slightly curved. Terminal branchlets are 6–35 mm long (Fig. 5A). The coenenchyme is granulose and brittle. Coenenchymal sclerites are of different forms: irregular spindles with acute or bifurcated ends, some are slightly curved (Fig. 6A), 0.14–0.21 mm long and 0.05–0.09 mm wide. Warty radiates and crosses are 0.13–0.15 mm long and 0.10–0.11 mm wide (Fig. 6B). Wart-clubs are 0.11–0.18 mm long and 0.05–0.085 mm wide at the expanded head being variable in form with a larger end expanded and covered with thorny warts and leaf-like projections (Fig. 6C). Coenenchymal sclerites are of various colours: orange, red, and darker (Fig. 5C).

The calyces are arranged all around the branches, not very close, slightly raised up to 0.5 mm tall as small mounds composed of eight marginal lobes with small polyp apertures at the summits (Fig. 5B). Thorny, irregular spindles and some wart-clubs appear often, around the calyx aperture. The anthocodial armature is well developed and composed of spiny spindles arranged in a collaret and points, 0.12–0.20 mm long and 0.032–0.056 mm wide (Fig. 6D); its flat rods are with small tubercles and scattered warts. Anthocodial sclerites are red and orange (Fig. 5C).

###### Remarks and comparison.

The calyces in this specimen are more separated and do not have swollen polyp apertures as in *P.arbusculatypica*. Verrill (1868b) mentioned a more flabellate colony but YPM 1787 is not flabellate having only a few branches. Verrill (1868b) did not provide data approximately the size of sclerites of this variety, uniquely pointing out that the sclerites resemble the ones of *P.arbusculatypica*. We found that the sclerites of the only specimen of this variety are smaller and similar to, the ones of *P.arbusculatypica*. Specimen YPM 8684 corresponds to several colony fragments belonging to *P.teres*, *P.arbuscula* and an undetermined species.

###### Distribution.

Tropical eastern Pacific: only reported from the type locality, Pearl Islands, Panamá.

##### Psammogorgia arbuscula var. pallida

Taxon classificationAnimaliaAlcyonaceaPlexauridae

Verrill, 1868

20BF18E2-B9E2-5819-A1C8-BA5CB43625FE

Figures 7 , 8 , 9

Psammogorgia arbuscula var. pallida Verrill, 1868b: 415–416; Kükenthal 1919: 237; Harden 1979: 119.
Psammogorgia arbuscula pallida Kükenthal, 1924: 107.

###### Type material.

***Syntypes***: MCZ 729 (4916), YPM 1785a-b, dry, Pearl Islands, Panamá, F.H. Bradley, 1866–1867, no additional data.

###### Description.

**Figure 7.**
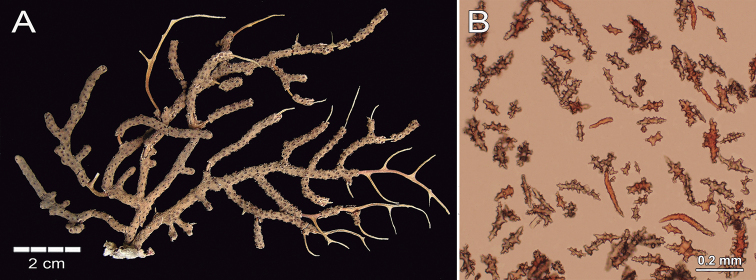
Psammogorgiaarbusculavar.pallida Verrill, 1868. MCZ 729 (4916). **A** Colony **B** coenenchymal and anthocodial sclerites.

**Figure 8.**
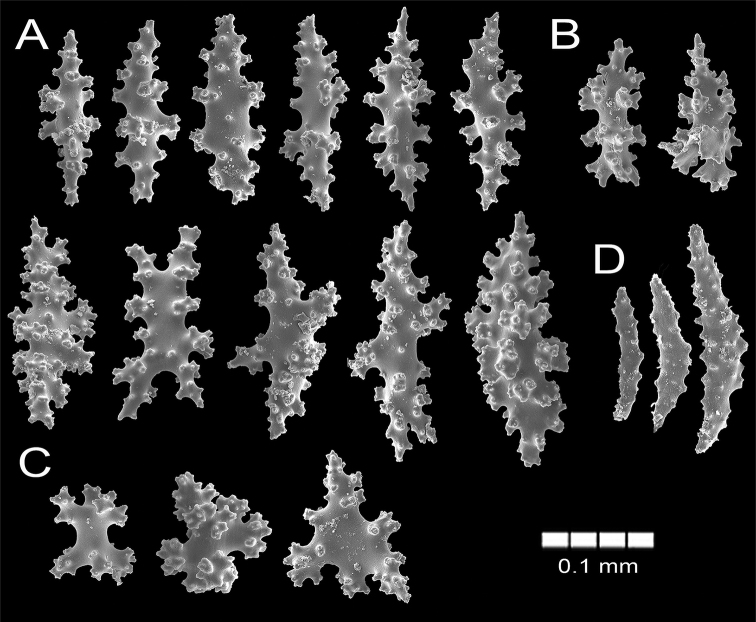
Psammogorgiaarbusculavar.pallida Verrill, 1868. MCZ 729 (4916) **A** spindles **B** wart clubs **C** radiates and crosses **D** anthocodial spindles.

The syntype MCZ 729 is a yellowish flabellate, 10.5 cm long and ~ 9 cm wide colony. Two main stems arise from a holdfast that is 1.6 cm in diameter nd devoid of polyps (Fig. 7A). The stems are less than 1 cm tall and 2.5 mm in diameter, subdividing irregularly in secondary branchlets of 2–3 mm diameter with rounded tips (Fig. 7A). The branchlets emerge at angles of 45–180° and spread irregularly in one plane. The colony branches up to 15 times. Terminal branchlets are up to 40 mm long (Fig. 7A). The polyps occur all around the branches, 1–1.5 mm apart on branchlets and 1.5–2.5 mm apart on the branches. The calyces are almost flat with a few being ~ 0.02 mm tall with oval or round polyp-apertures. Thorny, irregular spindles and wart-clubs occur around the calyx rim (Fig. 7B). The coenenchymal sclerites are mostly irregular tuberculate spindles (Figs 7B, 8A) with acute or bifurcated ends or combinations of both (Fig. 8A) with colours varying from pale pink to mostly colourless (Fig. 7B). These sclerites are 0.15–0.23 mm long and 0.04–0.09 mm wide. Wart clubs are 0.11–0.14 mm long and 0.05–0.06 mm wide with warts or with wide tubercles (Fig. 8B). Crosses various intermediate forms and radiates are 0.08–0.16 mm long and 0.07–0.12 mm wide with tubercles (Fig. 8C). The anthocodial armature is composed of slightly bent spiny orange-red sclerites arranged in a collaret and points, 0.11–0.23 mm long and 0.02–0.045 mm wide (Figs 7B, 8D).

**Figure 9.**
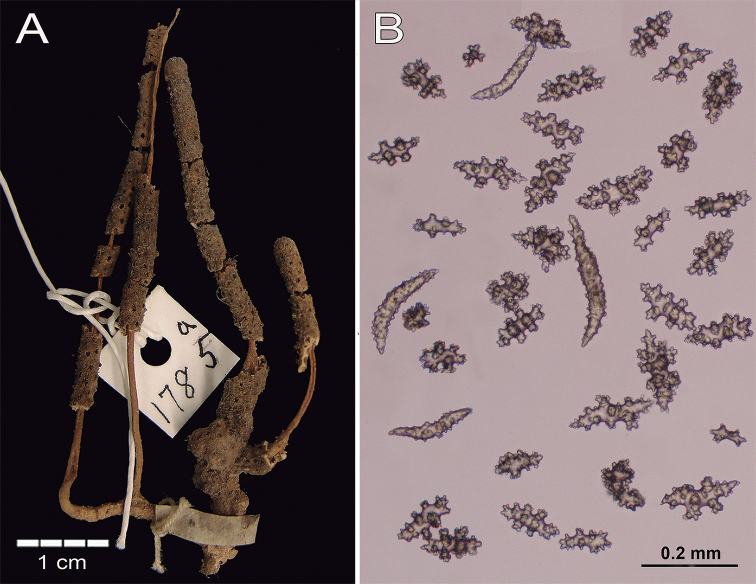
Psammogorgiaarbusculavar.pallida Verrill, 1868. YPM 1785 **A** colony **B** coenenchymal and anthocodial sclerites.

***The syntypes***YPM 1785a, b are two fragments of a lighter colour than MCZ 729 (Fig. 9B). The largest fragment is 5.6 cm long and composed of four branchlets with 2–3 mm in diameter; flat calyces all around the branches (Fig. 9A). All sclerites, including the anthocodials, are pale yellow to colourless. Sclerites from the syntypes are more ornamented than in specimen MCZ 729 but mostly colourless (Fig. 9B). The coenenchymal sclerites are mostly irregular, tuberculate warty spindles with acute or bifurcated ends or combinations of both; being 0.13–0.18 mm long and 0.035–0.08 mm wide. Clubs have a few warts or with wide tubercles being 0.11–0.16 mm long and 0.05–0.60 mm wide. Radiates have tubercles appearing in various intermediate forms, being 0.07–0.10 mm long and 0.05–0.08 mm wide. The anthocodial armature is composed of slightly bent spiny spindles arranged in collaret and points, 0.12–0.22 mm long and 0.025–0.043 mm wide.

###### Remarks and comparison.

Verrill´s material at MCZ includes two specimens, MCZ 729 (4916) and YPM 1785, both with similar sclerites but different in external morphology. One is a small colony and the other is a small fragment in bad condition. According to Verrill´s description (Verrill 1868b) the type material has a “corallum more or less flabelliform, branching dichotomously, branchlets round, sometimes as large as the main stem, usually smaller. Cells a little raised forming low verrucae”. However, Verrill did not measure the specimens. YPM 1785 is different from Verrill’s description while MCZ 729 (4916) matches some details of his description. Orange-red anthocodial sclerites are present in MCZ 729, as well as at the P.arbusculavar.pallida description by Verrill (1868b), in contrast with the colourless rods in specimen YPM 1785. Also, pale pink, colourless, and transparent coenenchymal sclerites match his description (Fig. 7B). In terms of sclerite sizes, Verrill’s description better matches MCZ 729 with larger sclerite sizes than the smaller YPM 1785 ones. As Verrill suggested, with this and *dowii* variety, we opt to keep P.arbusculavar.pallida as a variety.

###### Distribution.

Tropical eastern Pacific: only reported from the type locality at Pearl Islands, Panamá.

##### Psammogorgia fucosa

Taxon classificationAnimaliaAlcyonaceaPlexauridae

(Valenciennes, 1846), nomen dubium

1FF7F0E3-84E4-5E36-B9A1-58CB79085D77

Figures 10 , 11

Gorgonia fucosa Valenciennes, 1846: pl. 15
Plexaura fucosa Milne-Edwards & Haime, 1857: 154; Valenciennes 1855: 12.
Psammogorgia fucosa Verrill, 1868b: 417; 1870: 556–557; 1869: 427; Kükenthal 1919: 237; Kükenthal 1924: 107; Harden 1979: 118–119.

###### Type locality.

Mazatlán, México, Pacific coast (Valenciennes 1855).

###### Type material.

**Figure 10.**
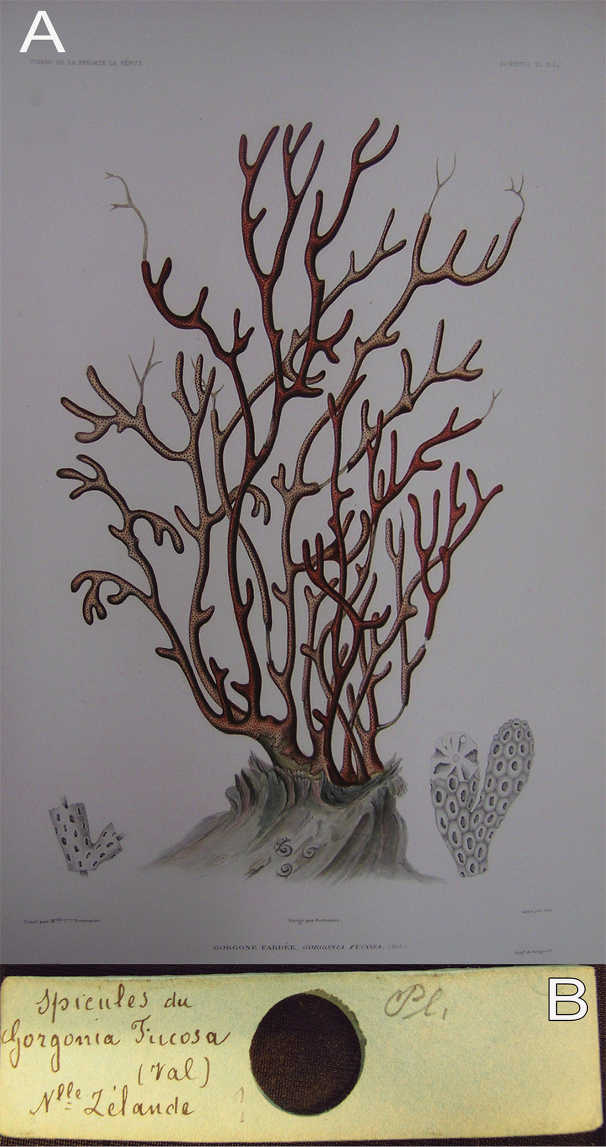
*Psammogorgiafucosa* Valenciennes, 1846 **A** original figure of the holotype, Valenciennes 1846: plate15 **B** microscopic slide with sclerites deposited at MNHN.

Plate 15, figured specimen (Valenciennes 1846). MCZ ¨spicules du *Gorgoniafucosa* sclerite slide from MNHN. Holotype figured. Valenciennes material from ‘Voyage autour du monde sur la frégate la Vénus, pendant les années 1836–1839’ expedition was deposited in the MNHN; however, the specimen was not found in the museum (M. Castelin, MNHN, pers. comm. March 2018). The description below is based on Verrill (1868b), Bayer (1958), the figured specimen of Valenciennes (1846: plate 15), and MNHN sclerite slide (Fig. 10B).

###### Diagnosis.

Colony dull reddish. Colonies bushy and irregularly dichotomous. Stems short and up to 12.5 mm in diameter. Branchlets up to 4.5 mm in diameter. Calyces flat, sparsely distributed all around the branches. Coenenchymal sclerites red, pink or colourless, mostly spindles up to 0.22 mm long; wart-clubs, up to 0.18 mm long; and warty radiates. Anthocodial spindles red, up to 0.21 mm long.

###### Description.

Valenciennes´ figured type was originally presented in natural size (Fig. 10A). Verrill (1868b) reported this dull reddish specimen to be 25.4 cm long and 22.8 cm wide, with the branches ~ 3.8 mm wide. Approximately five stems arise from the holdfast, the thicker being around 12.5 mm in diameter. The colony branches up to 12 times. The branches are irregularly dichotomous, emerging at angles of 45–120°, mostly ascending in parallel and bifurcating at distances of 12.5 to 50.8 mm. The end branchlets are mostly crooked, scarcely tapering and obtuse or clavate at the tips with a diameter of 3–4.5 mm. Branchlets tips are ~ 12.7 to 25.4 mm long (Fig. 10A). The calyces occur all around the branches but not close to each other (Fig. 10A).

**Figure 11.**
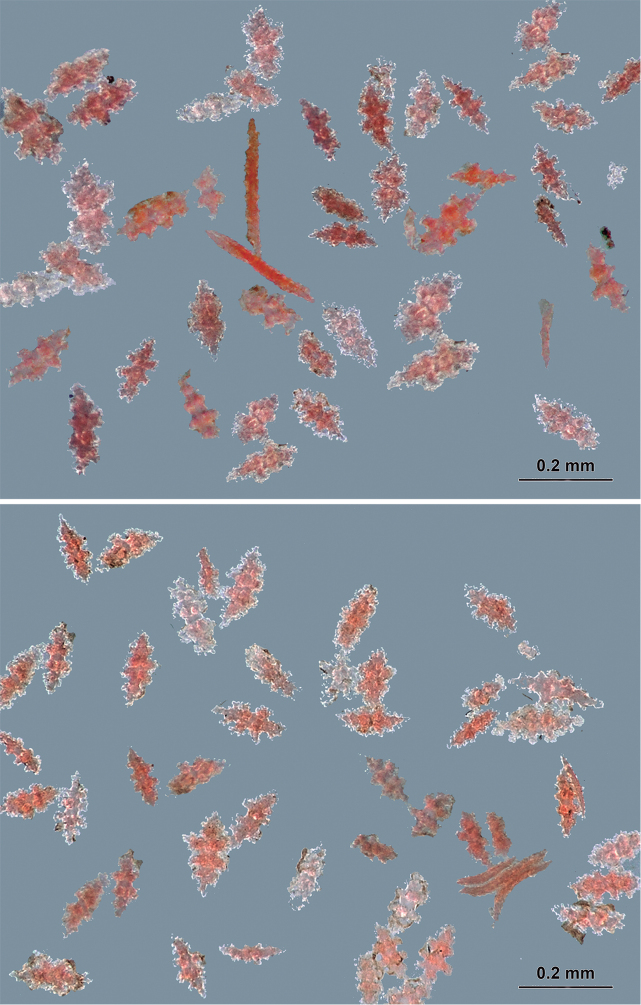
*Psammogorgiafucosa* Valenciennes, 1846. Coenenchymal and anthocodial sclerites, in stereomicroscope. Photographs: Jennifer Winifred Trimble IZ Curatorial Assistant, MCZ, 2018.

The coenenchymal sclerites vary remarkably in diversity of colour, size and form as Verrill has pointed out. Verrill found white, yellowish, light red, deep red and amethystine intermingled sclerites while we observed transparent, red and pink sclerites in the MNHN slide. The MNHN sclerites show a diversity of sclerites that is typical of the genus: mostly irregular warty spindles with acute, blunt or bifurcated ends, and several irregular forms (Figs 10B, 11). These sclerites measure 0.10–0.19 mm in length (reaching 0.22 mm according to Bayer (1858)) and 0.04–0.095 mm in width. Spindles commonly lack the naked median space as they are densely covered with warts. Few wart-clubs are found in the sample, 0.10–0.18 mm long and 0.04–0.06 mm wide at the expanded head. Radiates are densely covered by warts measuring 0.09–0.11 mm in length and 0.055–0.07 mm in width (Fig. 11). Anthocodial spindles are red, long, slender, and covered with small warts measuring 0.10–0.21 mm long and 0.01–0.02 mm wide (Fig. 11).

###### Remarks and comparison.

Verrill´s description of sclerites was based on the MNHN sclerite slide that was sent to him at the MCZ for analysis, probably by R.A. Kölliker (Bayer 1958). We have also analysed sclerites from the slide, showing details that are difficult to compare with those of other species. Though, we found larger sizes of the sclerites than sizes given by Verrill (1870), as also observed by Bayer (1958). This species is similar to *P.teres*, in many aspects (see analysis below). It is indeed possible that *P.fucosa* is a synonym of *P.teres*; however, without a specimen to examine we prefer to keep the status of *P.fucosa* as dubious.

###### Distribution.

Tropical eastern Pacific: only reported from the type locality at Mazatlán, México.

##### Psammogorgia gracilis

Taxon classificationAnimaliaAlcyonaceaPlexauridae

Verrill, 1868

7BA59E16-FC78-5DA9-8829-37EE56C2FFD8

Figures 12 , 13

Psammogorgia gracilis Verrill, 1868b: 417–418; Kükenthal 1919: 238; Kükenthal 1924: 108; Breedy & Guzman 2011: 29.
Heterogorgia gracilis Harden, 1979: 112–113.

###### Type material.

***Lectotype*** (designated herein): YPM 813a, dry, Pearl Islands, Panamá, F.H. Bradley, 1866–1867, no additional data.

***Paralectotype***: YPM 813b, dry small fragment, same data as the lectotype.

###### Type locality.

Pearl Islands, Panamá.

###### Diagnosis.

Colonies red, tall, flabelliform, branches subparallel and elongated. Stem a few centimetres long. Calyces close together and all around the branches. Calyces slightly raised and swollen with concentration of wart-clubs up to 0.25 mm long, and acute spindles around the calyx rim. Coenenchymal sclerites: irregular spindles with acute or bifurcated ends up to 0.20 mm long; warty and irregular radiates and some crosses. Anthocodial spindles up to 0.24 mm long in collaret and points arrangements. Sclerites red and orange or of lighter hues.

###### Description.

**Figure 12.**
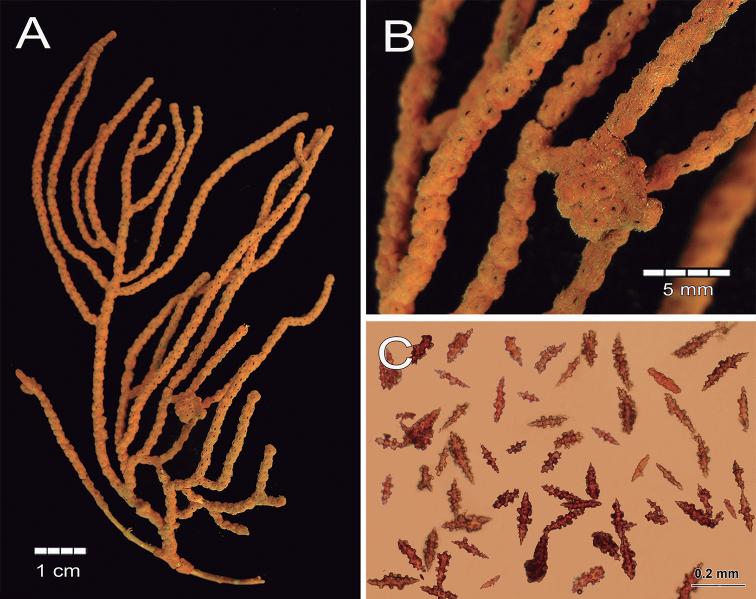
*Psammogorgiagracilis* Verrill, 1868. YPM 813a **A** colony **B** detail of branches showing a tumor **C** coenenchymal and anthocodial sclerites.

**Figure 13.**
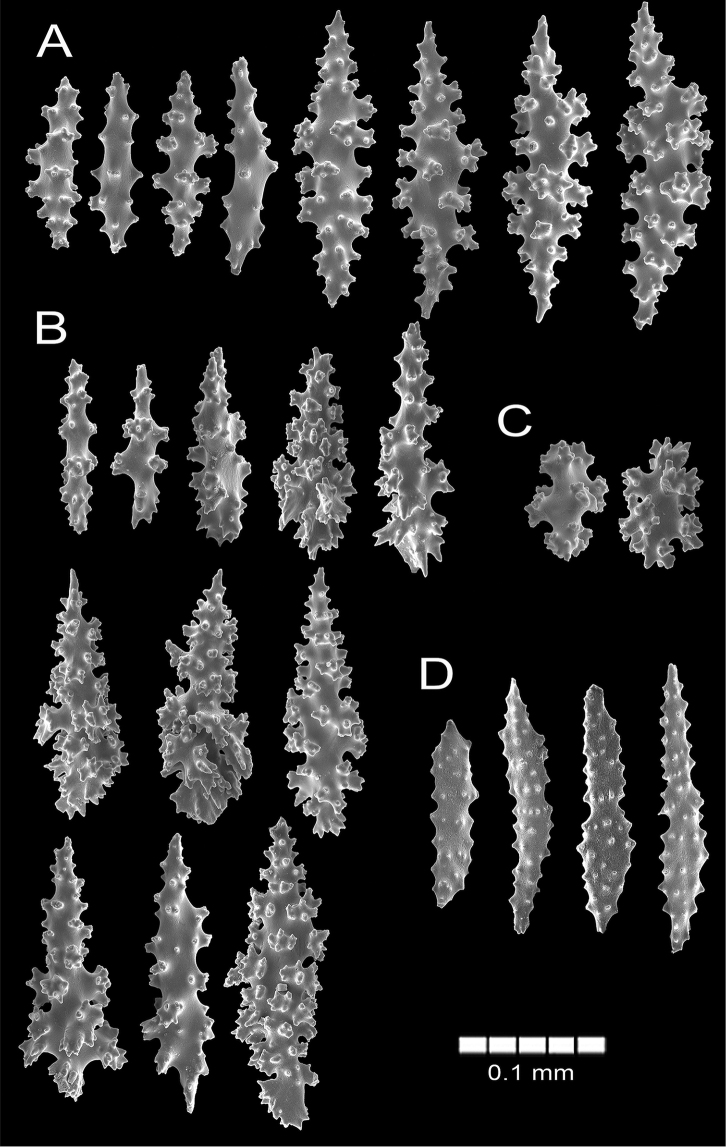
*Psammogorgiagracilis* Verrill, 1868. YPM 813a **A** spindles **B** wart clubs **C** radiates **D** anthocodial spindles.

The lectotype colony is red and 10 cm long and ~ 6 cm wide. Branching is irregularly dichotomous. The holdfast is absent. The branches emerge at angles of 45–90°, ascend parallel and slightly curve. The main branch of the colony is 2.5 mm in diameter subdividing into long, slender, ascending branchlets, 1.5–1.6 mm in diameter. Branches are round, slender, some extending up to 6.3 cm, undivided or bifurcating at the ends. Branches subdivide up to nine times while terminal branchlets are up to 60 mm long with rounded slightly tapered tips. (Fig. 12A). The calyces are densely arranged around the branches, slightly raised, swollen and are around 0.3 mm tall and 1 mm in diameter (Fig. 12B). The calyces have wart-clubs and some irregular spindles around the calyx rim and the base of the anthocodiae. Coenenchymal sclerites are red, orange or of lighter hues (Fig. 12A–C); including slim spindles with acute ends that may bifurcate (Figs 12C, 13A). Spindles are 0.12–0.24 mm long and 0.04–0.07 mm wide. Wart-clubs are 0.11–0.25 mm long and 0.04–0.08 mm wide at the expanded head (Fig. 13B). Warty radiates and crosses are 0.08–0.10 mm long and 0.055–0.07 mm wide (Fig. 13C); and some warty crosses up to 0.10 mm by 0.09 mm. Anthocodial armature is well developed with orange and pale yellow spiny spindles arranged in collaret and points (Fig. 12A–C), measuring 0.11–0.20 mm long and 0.03–0.04 mm wide (Fig. 13D) and flat spindles with small tubercles and scattered warts.

###### Remarks and comparison.

This species differs from the others by having long, slender and ascending branchlets, which are thinner than in the other species of the genus (Table 1). Verrill (1868b) pointed out the abundance of wart-clubs in this species when compared to *P.arbuscula* and *P.teres*. According to Verrill, the specimen he described was slender, flabelliform with subparallel and elongated branchlets, measuring12.7 cm long and 10.2 cm wide. The material left (YPM 813a) matches Verrill´s description and illustration. Therefore, we designate this specimen, YPM 813a, as the lectotype of *P.gracilis*.

###### Distribution.

Tropical eastern Pacific: only recorded at the type locality Pearl Islands, Panamá.

##### Psammogorgia hookeri

Taxon classificationAnimaliaAlcyonaceaPlexauridae

Breedy & Guzman, 2014

3803633F-E03B-5577-ADB2-739999D2ED9B

Figure 14

Psammogorgia hookeri Breedy & Guzman, 2014: 2–5.

###### Type locality.

Isla Gallán, Paracas National Reserve, Perú.

###### Diagnosis.

Colonies coral red, small, bushy, multiplanar and irregularly dichotomous. Coenenchyme granular. Coenenchymal sclerites: wide, irregular spindles with acute or bifurcated ends, and combinations of both; warty and irregular radiates, crosses and conspicuous star-like radiates. Colours of coenenchymal sclerites reddish, coral red, and lighter. Calyces prominent, swollen and closely placed. Thorny, irregular spindles, and wart-clubs around the calyx rim up to 0.16 mm long. Anthocodial spindles, thin and spiny, in collaret and points arrangements, yellowish, and pale pink in colour. We refer to Breedy and Guzman (2014) for a full description of the species.

###### Distribution.

**Figure 14.**
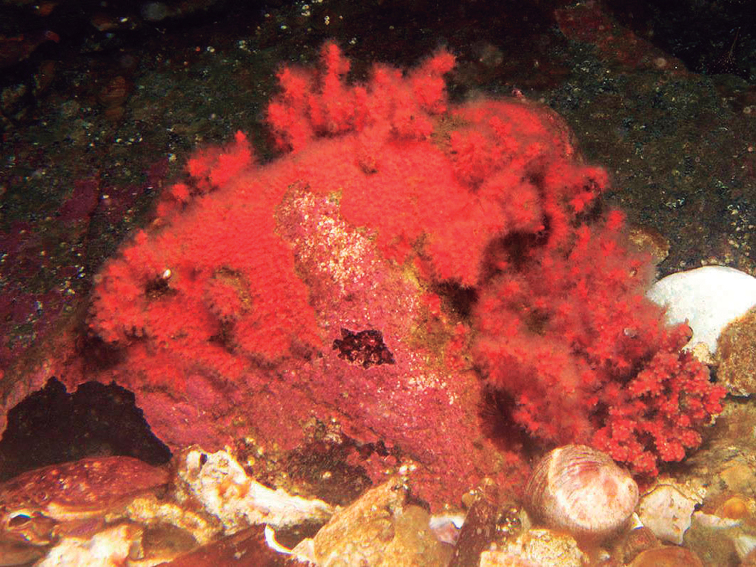
*Psammogorgiahookeri* Breedy & Guzman, 2014. *In situ* colonies at Isla San Gallán, Paracas National Reserve, 25 m deep. Photograph: Yuri Hooker (UPCH).

This species has only been reported for Perú, Isla Gallán, Paracas National Reserve at 25 m depth, and from Bahía Independencia at unknown depth (Fig. 14).

##### Psammogorgia pax

Taxon classificationAnimaliaAlcyonaceaPlexauridae

Breedy, Guzman, Murillo & Vargas, 2020

50E8E039-73EF-59CD-805E-31E8C314EA43

Psammogorgia pax Breedy et al., 2020: 5–7.

###### Type locality.

Hannibal Bank, Gulf of Chiriquí, Pacific Panamá.

###### Diagnosis.

Colonies white, flabellate and branching in one plane, profuse irregularly dichotomous with occasional anastomosis. Calyces slightly raised, not close together with spiny lobes around polyp apertures. Thorny, irregularly-shaped spindles, and wart clubs around the calyx rims; wart clubs up to 0.26 mm long. Coenenchyme granular. Coenenchymal sclerites white, irregular spindles with acute or bifurcated ends or combinations of both as well as warty and irregular radiates. Anthocodial spindles orange, thin and spiny, in collaret and points arrangements. We refer to Breedy et al. (2020) for a full description of the species.

###### Distribution.

This species has only been reported from its type locality in the upper mesophotic habitats of the Hannibal Bank at 63 m depth.

##### Psammogorgia teres

Taxon classificationAnimaliaAlcyonaceaPlexauridae

Verrill, 1868

FF157F0D-D21C-5A17-B821-7F232170016A

Figures 15 , 16

Psammogorgia teres Verrill, 1868b: 416–417; Hickson 1915: 554; Kükenthal 1919: 237–238; Kükenthal 1924: 108; Harden 1979: 120.

###### Type material.

***Lectotype*** (designated herein). YPM 1556b, dry, Pearl Islands, Gulf of Panamá, Panamá, F.H. Bradley, 1866–1867, no additional data.

***Paralectotype*.**YPM 1556a, c, same data as the lectotype.

###### Type locality.

Pearl Islands, Panamá.

###### Diagnosis.

Colonies red or orange when preserved but brighter when alive. Colonies bushy and branch laterally and irregularly dichotomous. Stems vary from few millimetres up to 5 cm long, and 6 mm in diameter. Holdfasts encrusting with thin coenenchyme, often with polyps. Calyces flat, sparsely distributed all around the branches. Calyces with thorny, irregular spindles and wart-clubs around the calyx rim. Coenenchyme compact. Coenenchymal sclerites red, orange or colourless, mostly irregular warty spindles with acute or bifurcated ends and asymmetrical forms with prominent warty tubercles up to 0.20 mm long; wart-clubs with wide heads, up to 0.16 mm long; warty radiates and crosses. Anthocodial spindles pale yellow or colourless, flat or spiny, up to 0.26 mm long and in collaret and points arrangements. Coenenchymal sclerites red, orange and colourless, anthocodial rods pale yellow and colourless.

###### Description.

**Figure 15.**
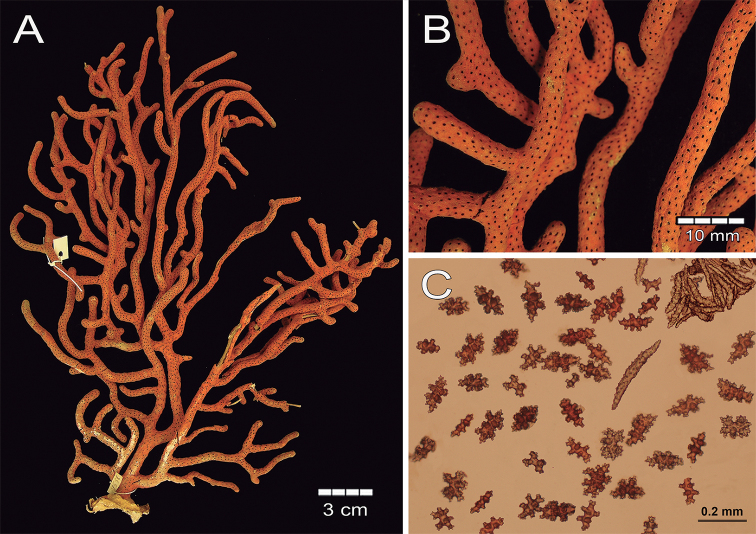
*Psammogorgiateres* Verrill, 1868. YPM1556b **A** colony **B** detail of branches **C** Coenenchymal and anthocodial sclerites.

**Figure 16.**
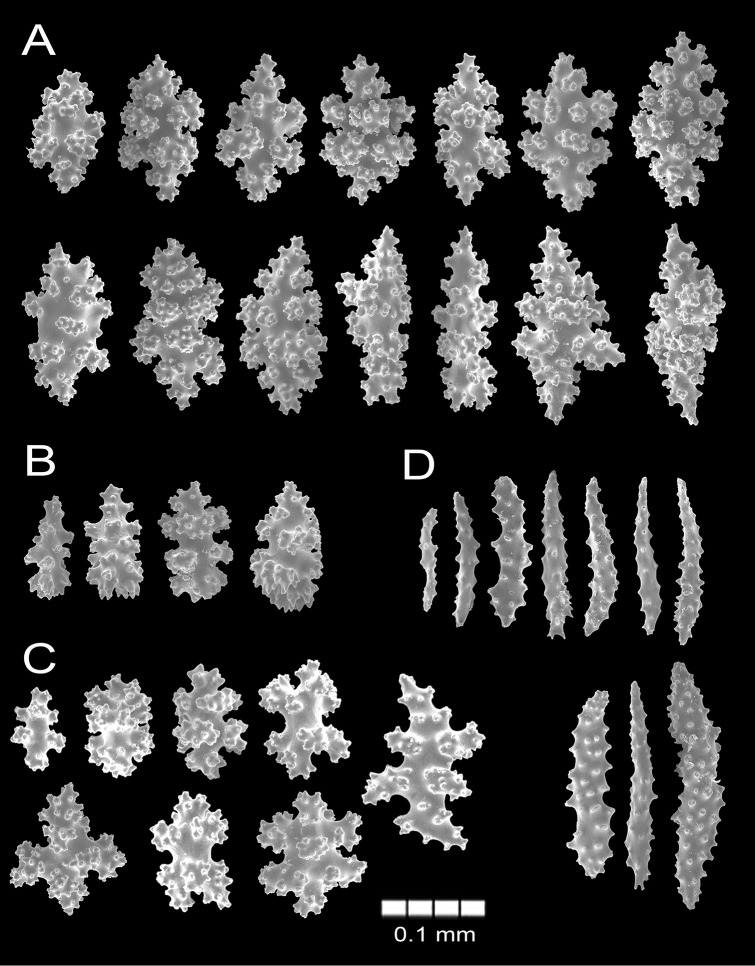
*Psammogorgiateres* Verrill, 1868. YPM1556b **A** spindles **B** Wart clubs **C** Radiates and crosses **D** anthocodial spindles.

The lectotype is a red orange dry colony, which was brighter when alive (Verrill 1868b), with 25 cm long and 20 cm wide (Fig. 15A, B). The colony is bushy and laterally branched with an irregularly dichotomous pattern which branches up to 12 times (Fig. 15A, B). The stem is 5 mm long and is 6 mm in diameter, arising from an oval holdfast with around 3.1 cm in diameter that bifurcates in two main branches. These branches are 5–6 mm thick at the base diminishing toward the tips to branchlets of around 3 mm in diameter (Fig. 15A). The branches emerge at angles of 45–90°, ascending mostly parallel to each other and bifurcating the same way. Branchlets are mostly perpendicular to the branch of origin and slightly curved. Terminal branchlets are 5 to 60 mm in length (Fig. 15A). Calyces occur all around the branches, being flat and with a polyp rim 0.4–1.0 mm in diameter and, mostly separated between each other by 0.5–4.0 mm with an average of 3.5 mm (Fig. 15B). Calyces have concentration of thorny, irregular spindles and wart-clubs appearing usually around the calyx rim. The coenenchyme is compact with a finely granulated surface. The coenenchymal sclerites are very variable in size and form, being red, orange or colourless and mostly composed of irregular warty spindles with acute or bifurcated ends, and some asymmetrical forms with prominent warty tubercles (Figs 15C, 16A). Spindles are 0.11–0.20 mm long and 0.07–0.12 mm wide. Wart-clubs have wide leafy heads and are 0.07–0.16 mm long and 0.045–0.10 mm wide (Fig. 16B). Warty radiates are 0.07–0.13 mm long and 0.06–0.09 mm wide (Fig. 16C); and some crosses, 0.95–0.11 mm by 0.08–0.11 mm (Fig. 16C). The anthocodial armature is well developed and composed of pale-yellow to colourless spiny spindles and flat warty sclerites arranged in collaret and points, measuring 0.13–0.24 mm in length and 0.02–0.04 mm in width (Figs 15C, 16D).

###### Remarks and comparison.

While the largest anthocodial sclerite measured in the lectotype was 0.24 mm long, Verrill (1886b) mentioned a slightly larger length of 0.26 mm. This is in accordance to the anthocodials of other specimens revised in this study. The syntype YPM1556b closely fits Verrill´s (1868b) description of the colony and the sclerites. For this reason, we designate this as the lectotype to clearly establish the species identity.

*Psammogorgiateres* has a colony morphology similar to that of *P.fucosa* (Table 1), but it has different sclerite sizes and colours in comparison with the type’s sclerite slide (Table 2). In *P.teres*, spindles and wart-clubs are shorter while anthocodials and radiates are larger than in *P.fucosa* (Table 2). Anthocodials of *P.fucosa* are red but colourless in *P.teres*, which is a diagnostic feature of this species.

In comparison with *P.arbuscula* and *P.gracilis*, *P.teres* differs in the external morphology represented by colonies with thicker branches and flat calyces; and relative abundance and sizes of sclerites (Tables 1, 2).

###### Distribution.

The species occurs in Pearl Islands, Panamá (type locality) and also in in the Chiriquí Gulf, Panamá. However, the species presents a wider regional distribution in the tropical eastern Pacific. It was sampled by us, along the Pacific coast of Costa Rica, El Salvador, Nicaragua, and Ecuador, and encountered in collections from the Pacific coasts of México and Colombia.

## Conclusions

The genus *Psammogorgia* comprises six species and two varieties belonging to two morphological species-groups: *Psammogorgiaarbuscula* group consisting of *P.arbuscula*, *P.gracilis*, *P.hookeri* and *P.pax*. and the *Psammogorgiateres* group consisting of *P.teres* and *P.fucosa*. We have explored and collected *Psammogorgia* species in Costa Rica, El Salvador, Nicaragua and Panamá, and have revised collections from Colombia, Ecuador, México and Perú. We found *P.arbuscula* and *P.teres* from these localities; nonetheless, *P.gracilis* and the two varieties of *P.arbuscula* were not found as additional records. Regarding *P.digueti*, after our analysis of a specimen in the MNHN we conclude that it belongs to a different genus and its status has to be revised. Lastly, *P.hookeri* and *P.pax* seem to be endemic to their regions, the first one from Perú and the other from mesophotic habitats off the Pacific coast of Panamá. However, without more explorations and further records, the geographic distribution and species richness of *Psammogorgia* is incomplete.

### Key to the valid species of the genus *Psammogorgia* Verrill, 1868 reported from the tropical eastern Pacific

**Table:** 

1	Colony white. Calyces slightly raised. Deep water species (> 60 m)	***P.pax***
–	Colony red or of different hues of red. Calyces prominent to flat. Shallow water species (< 40 m)	**2**
2	Calyces prominent with swollen polyp apertures	**3**
–	Calyces flat without swollen polyp apertures	**5**
3	Colony coral red. Wart clubs < 0.16 mm in length. Star-like radiates present in coenenchyme	***P.hookeri***
–	Colony red or dark red. Wart clubs > 0.16 mm in length. Star-like radiates absent from coenenchyme	**4**
4	Branch diameter > 2 mm. Anthocodial spindles > 0.20 mm in length	***P.arbuscula***
–	Branch diameter < 2 mm. Anthocodial spindles < 0.20 mm in length	***P.gracilis***
5	Terminal branchlets long (> 25 mm). Anthocodial spindles pale yellow to colourless and >0.20 mm in length	***P.teres***
–	Terminal branchlets long (< 25 mm). Anthocodial spindles red and < 0.20 mm in length	***P.fucosa***

## Supplementary Material

XML Treatment for Psammogorgia

XML Treatment for Psammogorgia arbuscula

XML Treatment for Psammogorgia arbuscula var. dowii

XML Treatment for Psammogorgia arbuscula var. pallida

XML Treatment for Psammogorgia fucosa

XML Treatment for Psammogorgia gracilis

XML Treatment for Psammogorgia hookeri

XML Treatment for Psammogorgia pax

XML Treatment for Psammogorgia teres
